# Poor Motor Competence Affects Functional Capacities and Healthcare in Children and Adolescents with Obesity

**DOI:** 10.3390/sports12020044

**Published:** 2024-02-01

**Authors:** Matteo Vandoni, Luca Marin, Caterina Cavallo, Alessandro Gatti, Roberta Grazi, Ilaria Albanese, Silvia Taranto, Dario Silvestri, Eleonora Di Carlo, Pamela Patanè, Vittoria Carnevale Pellino, Gianvincenzo Zuccotti, Valeria Calcaterra

**Affiliations:** 1 Laboratory of Adapted Motor Activity (LAMA)—Department of Public Health, Experimental Medicine and Forensic Science, University of Pavia, 27100 Pavia, Italy; matteo.vandoni@unipv.it (M.V.); luca.marin@unipv.it (L.M.); alessandro.gatti08@universitadipavia.it (A.G.); ilaria.albanese01@universitadipavia.it (I.A.); pamela.patane01@universitadipavia.it (P.P.); 2 Department of Research, ASOMI College of Sciences, 2080 Marsa, Malta; direttore@asomi-osteopatia.com; 3 Department of Sport and Exercise Science, LUNEX International University of Health, Exercise and Sports, 50, Avenue du Parc des Sports, 4671 Differdange, Luxembourg; cavallo.caterina@stud.lunex-university.net; 4 Pediatric Department, Buzzi Children’s Hospital, 20154 Milan, Italy; roberta.grazi@unimi.it (R.G.); silvia.taranto@unimi.it (S.T.); eleonora.dicarlo@unimi.it (E.D.C.); gianvincenzo.zuccotti@unimi.it (G.Z.); valeria.calcaterra@unipv.it (V.C.); 5 Industrial Engineering Department, University of Tor Vergata, 00133 Rome, Italy; 6 Department of Biomedical and Clinical Science, University of Milan, 20157 Milan, Italy; 7 Department of Internal Medicine, University of Pavia, 27100 Pavia, Italy

**Keywords:** motor performance, motor impairments, obesity, youth, physical activity level

## Abstract

Background: From a young age, children learn different motor skills known as fundamental motor skills. The acquisition of these skills is crucial for the future development of context-tailored actions that could improve adherence to physical activity (PA) practice. Motor competence and function deficits have been associated with pediatric obesity. We reviewed the literature data regarding motor competence in pediatrics and impaired motor performance in children and adolescents with obesity. Methods: We assessed the abstracts of the available literature (*n* = 110) and reviewed the full texts of potentially relevant articles (*n* = 65) that were analyzed to provide a critical discussion. Results: Children and adolescents with obesity show impaired motor performance, executive functions, postural control, and motor coordination. Children’s age represents a crucial point in the development of motor skills. Early interventions are crucial to preventing declines in motor proficiency and impacting children’s PA and overall fitness levels. Conclusions: To involve children, the PA protocol must be fun and tailored in consideration of several aspects, such as clinical picture, level of physical fitness, and motor skills. A supervised adapted exercise program is useful to personalized PA programs from an early pediatric age.

## 1. Introduction

Motor competence (MC) is a general expression that describes several terms used in the literature (i.e., motor proficiency, motor performance, fundamental movement/motor skills, motor ability, and motor coordination) to evaluate goal-directed human movement [1,2,3,4]. Motor development in children integrates both fine and gross motor coordination abilities. Fine motor coordination involves smaller muscle groups (e.g., hand and finger movements) and eye–hand coordination for the performance of fine tasks; conversely, gross motor coordination involves the use of bigger muscle groups (e.g., torso and legs) to accomplish various tasks such as walking and throwing [3].

From a young age, children learn different motor skills known as fundamental motor skills (FMS). The FMS are divided into two domains: locomotor skills that involve body movement in the space such as running, skipping, and hopping [5]; and object-control skills that consist of objects manipulation such as throwing, catching, and kicking [5].

The acquisition of these skills is crucial for the future development of context-tailored actions that could improve adherence to physical activity (PA) practice [6]. Indeed, previous studies have shown the existence of strong links between motor competence and PA, highlighting the importance of the latter in maintaining a healthy weight alongside supporting correct motor development in children [2,3]. The World Health Organization (WHO) always underlines that PA and active lifestyle promotion should be prioritized to prevent health diseases since exercise is proven to improve cardiovascular fitness and modulate inflammatory processes in all populations [4]. However, the higher levels of sedentary behavior among children and adolescences reported in the recent literature have led to a higher risk of weight gain, which is reflected in the steep increase in obesity prevalence and incidence in youth [1,4].

Motor competence and function deficits have been associated with pediatric obesity [1,2,3,4]. The global increase in the prevalence of childhood obesity is affecting children’s health and development [7]. Obesity is associated with various dysfunctions of the organism such as musculoskeletal problems. Moreover, children with overweight and obesity are less physically active than their healthy weight peers, showing lower levels of PA adherence and practice [8,9,10]. Pediatric patients with obesity show a reduced level of body control and functional abilities due to the excess in fat mass. Consequently, this condition impairs their ability to perform motor skills and take part in PA, finally resulting in delayed FMS development [11]. The abovementioned observations should be considered when designing an intervention to promote PA and healthy habits in the pediatric population with obesity.

In the present study, we reviewed the literature data regarding motor competence in pediatrics and impaired motor performance in children and adolescents with obesity.

## 2. Materials and Methods

**Figure 1:**
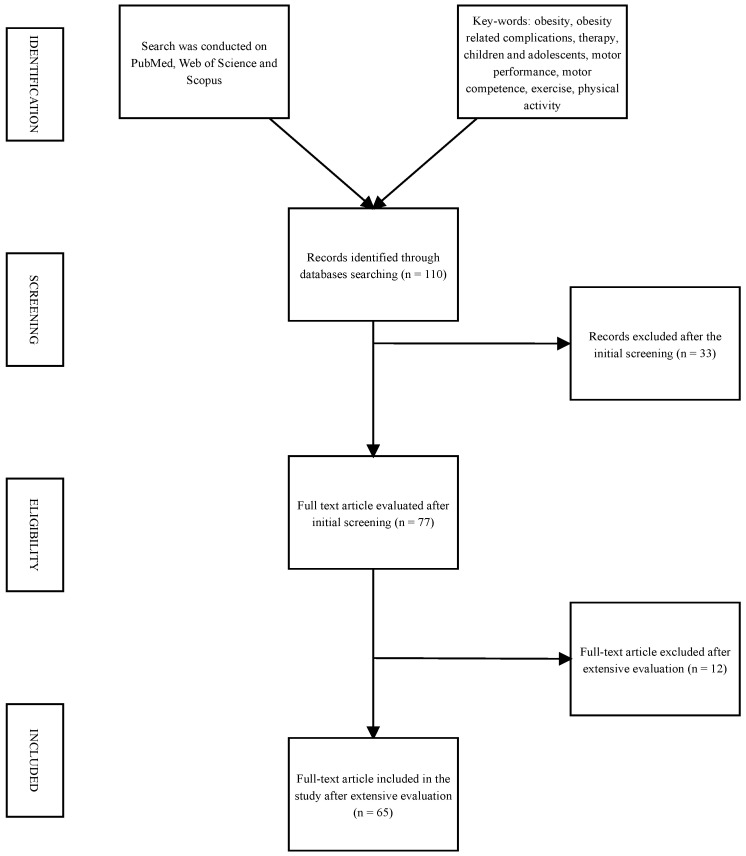
Flow chart of the study procedure.

We presented a narrative review of the literature [12], showing a not strictly systematic summation and analysis of the available literature on pediatric obesity and impaired motor competence and performance. To refine the scope of our overview, we established a set of inclusion criteria: articles published in the last twenty years in the English language; and original scientific papers, clinical trials, meta-analyses, and reviews published on a specific topic. Case reports, case series, and letters were excluded. The authors assessed the abstracts of the available literature (*n* = 110) and reviewed the full texts of potentially relevant articles (*n* = 65), which were analyzed to provide a critical discussion; the reference list of all articles was checked to identify relevant studies. The research terms adopted (alone and/or combined) are obesity, obesity related complications, therapy, children and adolescents, motor performance, motor competence, exercise, and physical activity. PubMed, Scopus, and Web of Science were used as databases for research purposes. A flow chart explaining the procedures of the study is shown in Figure 1. The contributions were independently collected by V.C.P., A.G., R.G., I.A., S.T., E.D.C, P.P., and C.C, and critically analyzed and discussed with D.S., V.C., M.V., and L.M. The resulting draft was critically revised by V.C., M.V., L.M., and G.Z. The final version was then recirculated and approved by all.

## 3. Childhood Obesity

Recent studies have included childhood obesity among the most important public health problems worldwide. Estimates show that more than 340 million children and adolescents aged between 5 and 19 years are either overweight or live with obesity, recording an alarming global increase in the prevalence of obesity that reached 18% in 2016 from 4% in 1975 [7]. There are, nowadays, nearly 50% more children under the age of 5 who are living with obesity [13].

The World Obesity Federation, in 2012, reported that alongside American regions that consistently hold the highest prevalence values worldwide (30% prevalence of overweight-to-obesity status), European-based pediatric cohorts have now reached alarming levels; data collected between 2015 and 2016 by the WHO European Childhood Obesity Surveillance Initiative (COSI) showed a prevalence of overweight and obesity of 20–40% [14,15].

The fundamental cause driving the trend of childhood obesity relies on an energy imbalance between calories consumed (due to increasing use of sugar-sweetened beverages, high glycemic foods, and foods containing excess fat) and calories expended (due to reduced levels of PA and increasing sedentary behavior such as use of television, computers, and video-games). The risk factors leading to the development of childhood obesity have been associated with parental feeding styles and parental obesity, psychosocial and emotional distress, sleep quality, and medications (e.g., antipsychotic and antiepileptic drugs). Other crucial factors include birth size, catch-up growth, breast-feeding status, and adverse life experiences. Heritable factors are responsible for 30–50% of the variation in adiposity, most commonly with a polygenetic etiology and, in less than 1% of the cases, with a single gene defect. Endocrine dysfunctions responsible for a gain in body weight are identified in less than 1% of children and adolescents with obesity and include endogenous or exogenous glucocorticoid excess, hypothyroidism, growth hormone deficiency, and pseudohypoparathyroidism type 1a (Albright hereditary osteodystrophy) [16].

Together with the increasing prevalence of obesity, the prevalence of associated comorbidities causing obesity-related diseases are also rising and have an important impact on our society [17,18]. As reported in Table 1, patients with obesity may experience various harmful disease-related health effects, including metabolic disorders, such as insulin resistance, hyperglycemia, type 2 diabetes mellitus, and dyslipidemia; alterations in cardiac structure and function, such as hypertension and endothelial dysfunction [19,20,21,22]; respiratory comorbidities, such as obstructive sleep apnea syndrome (OSAS) [23] and obesity hypoventilation syndrome (OHS); and gastrointestinal [24,25,26], endocrinological [27], neurological [28], dermatological [26,29], orthopedic [30,31,32,33], renal, and nutritional [34,35] complications. Lastly, psychosocial consequences of obesity may also occur. Finally, obesity has also been associated with increased risk of developing cancer during adulthood, especially breast cancer, colorectal cancer, leukemia, and Hodgkin lymphoma [36].

Dietary interventions might consist of dietary advice or structured plans associated, in some cases, with energy restriction. The first step of the educational process consists of the assessment of the patient’s and their family’s eating behavior, preferably through a food diary, and then providing dietary advice such as intake of five meals a day including an adequate breakfast; limitation of portions; increased intake of fibers, fruits, and vegetable; avoidance of food with high caloric density and poor of nutrients; encouraging family meals [39,40,41,42,43]. In line with the Dietary Guidelines for Americans [44], the advice extends to reducing the frequency of fast food consumption. When opting for fast food, the guidelines suggest choosing healthier side dishes, such as soup or fruit salad, instead of fries. The guidelines also suggest opting for lean protein sources, such as white meat, legumes, or tofu, instead of high-fat options such as sausages and fried meat. Further, they emphasize switching from highly processed foods to whole, nutrient-rich options such as fruits, vegetables, whole grains, nuts, and seeds.

In addition, the guidelines call for replacing sugary drinks with healthier alternatives such as water, low-fat or fat-free milk, or fortified non-dairy beverages. To reinforce these dietary choices, strategically, the guidelines recommend placing nutritious foods and beverages within reach and sight, while keeping high-calorie options out of sight or avoiding them altogether; for example, they suggest replacing the cookie jar with a bowl of fruit to encourage healthier snacking habits [44].

To increase its effectiveness, the recommendations advise combining physical exercise with diet.

Exercise is a non-pharmacological intervention that can increase energy expenditure, improve cardiovascular fitness, delay comorbidities, and modulate inflammation associated to obesity [2]. It might consist of at least 60 min a day of mainly aerobic and at least moderate-intensity exercises and the practice of resistance activity at least three times a week [45,46]. Older children should be encouraged to participate in structured sports after school such as football, swimming, and tennis; while younger children tend to prefer unstructured PA such as outdoor play [16]. Since children and adolescents affected by obesity often experience difficulties in exercising, the program might be personalized considering children’s abilities and baseline physical condition. In cases of severe obesity, it would be preferable to undertake exercises that do not involve significant impact or weight on the hips, legs, and feet. Previous studies have demonstrated that enjoyment plays a major role in increasing intrinsic motivation to start and maintain adherence in exercising [47]. Hence, in order to guarantee temporal continuity, the chosen exercise program should also be fun and appreciated by the child, and frequency, duration, and volume should be tailored according to the child’s preferences.

To increase daily energy expenditure, it is recommended to reduce sedentary behavior, such as television viewing or time spent internet surfing, and active video games, always provided under parental control, should be preferred to static ones. Also, sleep intervention, such as improvement of sleep hygiene, seems to be effective in weight loss, especially in pre-school age children [48].

To fulfill a greater adhesion to PA and diet, cognitive and family behavioral treatments are recommended. The most used cognitive behavioral techniques are contingency training, goal setting, stimulus control (through environment control), and self-monitoring (through PA and food diaries) [49]. Family behavioral techniques consist of changes in the whole family lifestyle and rely on the active participation of parents and other family members.

In cases of suboptimal reduction of BMI through lifestyle intervention or in selected cases affected by complications, pharmacological therapy could be administered.

An anti-obesity treatment approved by most regulatory agencies in patients aged between 12 and 18 years is liraglutide, which acts as an agonist of glucagon-like receptors, reducing appetite and slowing gastric motility. As demonstrated by the trial conducted by Kelly et al., the use of liraglutide, associated with lifestyle therapy, led to a significantly greater reduction in BMI SDS than the use of placebo associated with lifestyle therapy, without a significant reduction in cardiovascular risk [50]. Another pharmacological therapy that seems to induce weight loss and favor behavioral changes is orlistat, a tetra-hydro-liptinite reversible inhibitor of gastric and pancreatic lipases [51,52,53]. Semaglutide and the combination of phentermine and topiramate, recently tested in adolescence through clinical trials, led to a significant BMI and weight reduction. In view of these promising results, FDA recently approved the combination of phentermine and topiramate in the treatment of obesity in adolescents with an initial BMI in the 95th percentile or greater, standardized for age and sex [54,55].

Bariatric surgery represents the ultimate-stage therapy in the case of patients with severe obesity resistant to every non-chirurgic therapy. Surgical treatment is able to improve complications related to obesity, to reduce cardiometabolic risk factors, and to ameliorate muscle–skeletal pain and mobility [50,56,57,58,59].

## 4. Motor Competence in Children

Children who are not sufficiently efficient in FMS have limited opportunities to engage in PA practice, according to Clarke et al. [6]. It is widely assumed that FMS are “naturally” learnt by children, while Robinson and colleagues [60,61,62,63] showed that children who are taught motor skills by specialists show greater improvements in MC than children who engage in free play. Moreover, Stodden et al. [64] suggested that children’s PA participation might improve motor skills competence development due to higher promotion of neuromotor stimulation [65,66,67]. The extent of MC development is related to the differences in the experiences provided to children, including several factors such as environment, presence of structured physical education, socioeconomic status, parental influences, climate, etc. [68,69,70]. In addition, Barnett et al. [71] suggested that variables such as age, gender, and weight status affect MC, while Queiroz et al. [72] found a positive modulation and improvement of motor skills (in addition to body mass index (BMI)) in the youth who had higher access to PA thanks to improved environmental contexts, sports facilities, or sports clubs. The current results underline the relation between PA practice and central nervous system development [73], in this context, MC evaluation acts as an indirect healthy growth indicator [71]. For these reasons, early interventions are required in order to prevent declines in motor proficiency and to impact children’s PA and overall fitness levels.

Furthermore, prior research reported that children who participated in sports and PA achieved greater levels of MC during childhood and adolescence and remained active into adulthood [74]. Tammelin et al. [75] underline the importance of MC development as a fundamental enhancer of PA levels and antagonist of obesity. Barnett et al. [76] found that object-control skills favor children’s participation/engagement in moderate-to-vigorous PA and organized PA (3.6% and 18.3%, respectively), supporting the relationship between MC and PA. Moreover, motor competence is impacted by both individual and environmental factors, relying on constructive interaction between nature and nurture. In fact, outdoor playing and the freedom to move in open spaces are a crucial contributor to motor development [77,78]. Nevertheless, motor competence does not develop naturally; it requires instruction and feedback, as well as opportunities for free play [77]. For these reasons, physical education teachers and trainers should perform some activities during their lessons and training outdoors. Moreover, to better improve motor competence in children, teachers and trainers should propose goal-oriented activities that include different motor stimuli (i.e., jumping the rope, walking on a balance beam, throwing balls, running, and moving sticks or bricks).

As mentioned above, MCs are acquired through exposure to context-specific situations and range from basic skills to sport-specific abilities, where the first ones are necessary in order to develop the latter.

Several instruments to assess the MC are used in children’s process- and product-oriented assessment and are mainly related to the context of the physical education curriculum [79]. Therefore, basic MC assessment is grade- and age-specific and product-oriented, during these focus should lie in achieving a successful performance of the movement goal rather than prioritizing the quality or quantity of movement execution [80,81,82,83]. In general, MC test batteries assess various movement characteristics belonging to higher-order locomotor dimensions, object-control, and stability, such as speed, accuracy, self-confidence, bilateral hand coordination, hand–eye coordination, hand–foot coordination, and/or static/dynamic balance (e.g., Henderson et al. [84]; Goodway et al. [85]). In addition, the quality of the performance or the outcome belonging to skill execution might be context-specific, consequently varying according to the environment in which the task is performed (Newell [86]; Sigmundsson et al. [87]).

Common basic MC assessments like the MOBAK-1-2 test [88,89] examine two competence areas, which are object movement (OM) and self-movement (SM). Examples include bouncing a ball along a corridor (for OM) or running in a given sequence (for SM). There are also process-oriented assessments such as the Test of Gross Motor Development, 2nd Edition [90], which is typically used in the United States (US), and product-oriented assessments such as the Körperkoordinationstest für Kinder [91], which is generally adopted in Germany and Europe. The TGMD-2 consist of a standardized test that evaluates locomotor and object-control skills in children aged from 3 to 10 years old, and, in particular, it measures the coordination of trunk and limbs during certain tasks where task performance and result are not strictly related [90,92]. The TGMD-2 is a reliable and valid tool with which to assess the fundamental gross motor coordination in children, and it helps trainers and teachers to objectively evaluate children’s abilities, to monitor them across time, and to implement specific PE programs when needed. The KTK is a reliable and low-cost battery field test [93] able to evaluate motor coordination in children between 5 to 14 years of age and consists of four standardized task assessments, namely, walking backward, jumping sideways, moving sideways, and hopping for height [94,95,96]. Recently, Novak et al. [97] proposed a shorter version of the standard KTK; the KTK3 has a strong correlation with the KTK scores (r = 0.98, *p* < 0.001). In particular, the KTK3 seems more suitable to sports and school settings. Indeed, the hopping for height task was excluded from the conventional testing battery since it was considered time-consuming and it exposed subjects to a higher risk of injuries (e.g., ankle sprain) than other sub-tests [97,98]. Also, the KTK3 administration time is approximately 10 min compared to the 20 min for its longer version (KTK) [97]. Then, there is also the test of MC (TMC) [99], which consists of four different tests: two fine motor tasks to assess manual dexterity; and two gross motor tasks involving dynamic balance. In all tasks, the performance measure is the time to completion in seconds. The participants have the possibility to practice the tasks before the actual test. The bricks handling task is used to quantify aspects of fine motor performance and includes placing bricks and building bricks. This test seems to validly measure the specificity of motor abilities in children aged from 7 to 8 years old. All the specific tests characteristics are shown in Table 2.

## 5. Impaired Motor Performance and Therapeutical Approach in Children with Obesity

### 5.1. Impaired Motor Performance

In the literature, it has been shown that body composition in children and adolescents is related to the normal development of motor performance and gross motor coordination; obesity can adversely affect them [104]. Preschool and school ages are crucial for learning gross motor skills [105]. These will be useful for the subsequent development of specialized motor sequences usable in sports [6].

Strength, bilateral and upper limb coordination, running speed, balance, and agility are just some examples of motor activities [106]. The development of motor skills occurs in early childhood, and their acquisition is linked to the physiological maturation of the neuromuscular system and determined by environmental factors [107].

Obese children show worse performance in locomotor skills than children of a normal weight due to some biomechanical limitations: an increase in compressive and shear forces on the capital femoral growth plate that can alter the femoral angle; a decrease in hip and knee flexion, resulting in greater stiffness during walking; an increase in the amount of both absolute and muscular force needed to move the additional mass [108].

Executive functions also develop during school age. It is a set of “higher order” cognitive processes (e.g., attention, motor output) that allow us to initiate, regulate, and carry out goal-directed behaviors. These functions at essentials for planning and creating strategies for problem solving [108].

Executive functions, motor performance, coordination, and postural control are altered in obese children and adolescents, as supported by the theory of developmental plasticity [109].

In these subjects, the greater fat mass to be supported and moved against gravity can lead to a worse execution of motor coordination tasks [109]. The problems are not exclusively related to moving a mass in excess against the gravity; this indicates a worse performance in fine motor precision and in the manual dexterity task, which could be due to difficulties with the integration and processing of sensory information [109].

In the literature, high BMI and increased fat mass accumulation have been associated with a higher probability of developing a coordination deficit [110,111,112].

Cairney et al.’s study reported that differences in BMI and waist circumference remained significant and have increased over time [110].

A review of Barros et al. [113] shows that gross motor coordination in children with obesity is worse than their normal weight peers [114,115,116,117,118,119]. The increase in body mass is related to the decrease in the ability to balance, as demonstrated by Abdelkarim [120]

In addition, while lower overall performance of movements is related to overweight [121], children living with obesity show mild motor difficulties [122]; overweight and obesity are related to a lower perception of their physical competence [123], as well as to poorer achievements in standing long jump performance, side jump, 20 m of speed back and forward running [124], and decreased motor skills [125].

Motor coordination is affected by environmental and biological factors; even gender can influence motor performance. Thus, gender-based activities facilitate the movement execution of certain items belonging to motor coordination abilities. On average, girls report worse results both in terms of sport practice and body perception. This translates into higher body dissatisfaction and higher perceived fat scores than boys [123].

Furthermore, the muscular system has been shown to exert retrograde control over the central nervous system, influencing motor behavior [113].

### 5.2. Therapeutical Proposal

In the literature, there is clear evidence that traditional exercise intervention promotes weight loss, metabolic health, and strength, which are essential for improving children’s motor competence [126].

The training program generally consists of various exercises focusing mainly on muscular strength and neuromuscular training to improve hip abductor and quadriceps muscles. Interventions aiming to strengthen quadriceps and hip muscles, with the addition of various neuromuscular exercises, may lead to improvements in the knees’ position in relation to hip and ankle joints during locomotion. The first training session, during which participants are asked to perform non-weight-bearing exercises, is mainly preparatory. The following sessions start to integrate weight-bearing exercises. The choice of the starting weight is generally settled based on participants’ perceived level of effort, which should be around 5–8 (out of 10) on the modified Borg rated perceived exertion (RPE) CR-10 scale. In the case of pain, the trainer should consider reducing the resistance or the frequency or the number of repetitions. For each exercise, participants should perform 10 repetitions for three series [127].

The training protocols listed in Table 3 refer to Lim et al. for quadriceps-strengthening exercises [128] and to Bennell et al. for the hip-strengthening exercises [129].

The proposed neuromuscular exercises refer to the protocol of Bennel et al. and consist of balance exercises in mono or bipodal support [129]. Exercises’ performance quality is critical; participants should focus on keeping their knee over the foot during movement execution. In addition, participants should avoid a knee flexion exceeding 30° to prevent high joint loading and pain. Progression can be achieved introducing unstable surfaces like foam mats or balance boards. Table 4 summarizes neuromuscular exercises [127].

The intervention program, moreover, as suggested by Sanchez-Lopez et al., can be based on play, using recreational activity as a way to improve the body composition of children with overweight or obesity. The plan consists of a nine-month intervention with a total of four sessions per week of a 90-min duration each. The sessions were fun and uncompetitive, structured into warming-up, main activity, and cool-down. The main activity included popular games and sports adapted to children’s needs. The activities were mainly based on aerobic capacities. Moreover, excessive jumping activities were avoided to prevent extreme joints loading [130].

Considering the link between a less-active lifestyle and childhood overweight and obesity, as well as poorer motor skill levels, PA plays an important role during children’s growth and motor development. Wrotniak et al. [130] reported that there is a lower chance for children with a lower level of motor skills to be physically active, who are more likely to engage in sedentary activities, which, consequently, may increase the risk of developing overweight and/or obesity. Moreover, the body weight in excess and fatness could also make movement performance more difficult, hindering the adequate development of motor skills in less-active children [130].

Song et al., in 2021 [131], provided a 16-week after-school physical fitness program in students with low physical fitness. In this study, conducted by Song et al. in 2021, 36 participants performed 50-min sessions three times per week. Each session consisted of a 5-min warm-up phase, different exercises based on four physical fitness skills (10 min each), and a 5-min cool-down phase. Compared to previous proposals, 10 min were dedicated to carrying out flexibility exercises (Table 5). An improvement in flexibility is shown to increase physical performance by stabilizing movements and reducing the risk of future injuries [131].

Based on what we reviewed, lower levels of motor competence can be regarded as both a precursor and/or a consequence of overweight or obesity, with lower PA levels playing a key role among the possible influencing mechanisms [114].

## 6. Conclusions

Our review underscores the great impact of obesity on children and adolescents, resulting in impaired motor performance, gross motor skills, and motor coordination. Recognizing the importance of different age stages throughout the course of motor skills development, we advocate for early intervention through enjoyable and individualized PA protocols. These interventions, carefully tailored based on clinical considerations, fitness levels, and motor skills, have been shown to be effective in preventing and reducing the impaired motor function associated with childhood obesity. We suggest supervised and individualized exercise programs from an early pediatric age, encouraging physical education teachers to incorporate structured outdoor activities. This multifaceted approach aims to improve children’s motor skills, promoting the foundation for a healthier and more active lifestyle.

## Figures and Tables

**Table 1:** Multisystemic complications of childhood obesity [23,24,25,26,27,28,29,30,31,32,33,34,35,36,37,38].

Organs	Complications
Cardiovascular system	Hypertension, dyslipidemia, impaired cardiac structure and function, atherosclerosis, cardiovascular events
Endocrine system	Insulin-resistance, pre-diabetes, type 2 diabetes mellitus, metabolic syndrome, polycystic ovary syndrome, and hyperandrogenism
Gastrointestinal system	Nonalcoholic fatty liver disease, nonalcoholic fatty liver, nonalcoholic steatohepatitis, and cholelithiasis
Respiratory system	Obstructive sleep apnea syndrome, obesity hypoventilation syndrome, and asthma
Renal system	Chronic kidney disease
Nutritional system	Vitamin D and iron deficiency
Musculoskeletal system	Slipped capital femoral epiphysis, genu valgum and varus, impaired mobility, musculoskeletal pain, and fractures
Oncological risk	Breast cancer, colorectal cancer, leukemia, and Hodgkin lymphoma
Dermatologic system	Acanthosis nigricans, hidradenitis suppurativa, intertrigo, and stretch marks
Neurologic system	Intracranial hypertension
Psychosocial sphere	Low self-esteem, distorted peer relationship, anxiety, depression, and disordered eating patterns

**Table 2:** Characteristics of MC assessment tools.

Test Batteries	Authors	Age Range and Country	Domains Tested	Subtests/Sub-Tests/ Test Items	Reliability	Test Evaluation Procedure
MOBAK-1-2	Herrmann et al. [88]	6–8 years; developed in Germany and Switzerland	Basic motor competencies and qualifications divided into the following: 1. Object movement 2. Self movement	1. Object movement: - Throwing - Catching - Bouncing - Dribbling 2. Self-movement: - Balancing - Rolling - Jumping - Running sideways [88]	NA	A maximum of eight points can be reached in each area of the motor competencies. Points are assigned according to the guidelines described by Herrmann et al. [88].
TGDM-2	Ulrich, 2000 [90]	3–10 years; developed in the USA and commonly used in Australia, Iran, Netherlands, Korea, Belgium, Brazil, and China	Overview of gross motor skills divided into the following: 1. Locomotor 2. Object control	1. Locomotor subtests: - Running - Galloping - Hopping - Leaping - Horizontal jumping - Sliding 2. Object control: - Striking a stationary ball - Stationary dribbling - Catching - Kicking - Overhand throwing	Interrater reliability for the total score, locomotor subscale, and object control subscale (ICC: 0.92–0.96) were all excellent [100]	Every test can be conducted two times, achieving five points maximum for every try; then, the scores obtained should be summed to obtain a raw skill score (run, gallop, hop, etc.). The skill scores add up to a raw subtest score (Locomotor; object control), which is converted to a standard score following Ulrich guidelines; then, subtest standard scores are combined and converted to an overall gross motor quotient.
KTK	Novak et al. [97] Vandorpe et al. [101]	5–14 years; the test battery was developed in Germany then used in Belgium, Portugal, Netherlands, Switzerland, Austria, Brazil, Costa Rica, Denmark, Finland, and the US	Sensory–motor integration capacities for fine and gross control and coordination of the body [90]	The test batteries consists of the following: - Walking backwards on different balance beams; - Moving sideways on boxes; - Hopping for height; - Jumping sideways with both feet together [101]	Test-retest reliability: - Walking backwards: r = 0.8 - Moving sideways r = 0.84 - Hopping for height: r = 0.96 - Jumping sideways: r = 0.95 Total score reliability: r = 0.97 [101]	The test scores are assigned based on the manual guidelines described by Kiphard and Schilling in 2007. Each score is transformed into a motor quotient according to gender and age parameters based on the performance of normally developed German children in 1974 [101].
KTK-3	Biino et al. [102]	5–14 years	Evaluation of fine and/or gross motor coordination skills [102]	KTK-3 is a shorter version of the KTK test battery and includes the following - Moving sideways - Walking backwards - Jumping Sideways	Test–retest reliability of the three tests are the same as described in the previous row	The test scoring procedure is the same as described in the previous row.
TMC	Sigmundsson et al. [99]	5–83 years; developed and commonly used in Norway	It is composed of four different tests that evaluate the following: 1. Fine motor tasks based on manual dexterity 2. Gross motor tasks based on dynamic balance	1. Fine motor tasks: - Placing bricks - Building bricks 2. Gross motor tasks: - Heel-to-to walking - Walking/running in slopes	The Cronbach’s alpha value for the standardized test items was 0.79, which can be considered as acceptable [99]	The tests are measured in seconds, and for all the items, a lower time indicates a better performance [99].
BOT—Short form	Bruininks et al. [103]	4–21 years; developed in the USA and also used in Taiwan	Comprehensive overview of fine and gross motor development in four motor areas divided into the following 1. Fine Manual control - Fine motor precision (two items) - Fine motor integration (two items) 2. Manual Coordination - Manual Dexterity (one item) - Upper Limb coordination (two items) 3. Body coordination - Bilateral Coordination (two items) - Balance (two items) 4. Strength and agility - Running speed and agility (one item) - Strength (two items)	1. Fine motor precision: drawing lines through paths—crooked; Folding paper 2. Fine motor integration: copying a square; copying a star 3. Manual dexterity: transferring pennies 4. Upper-limb coordination: dropping and catching a ball—both hands; dribbling a ball—alternating hands 5. Bilateral coordination: jumping in place—same sides synchronized; tapping feet and fingers—same sides synchronized 6. Balance: walking forward on a line; standing on a balance beam—eyes open 7. Running speed and agility: one-legged stationary hop 8. Strength: knee push-ups; sit-ups.	Test–retest reliability in healthy children for all the mentioned items is above 0.70 [103]	In the BOT-2 test, raw scores must be transformed. The initial test results are converted into points ranging from 2 to 13; then, when all the points are added together, the total result is obtained [103].

MOBAK 1-2 = Motorische Basiskompetenzen; KTK: Körperkoordinationstest für Kinder; TGDM-2: Test of Gross Motor Development, 2nd Edition; TMC: Test of motor competences; BOT-short form: Bruininks–Oseretsky Test of Motor Proficiency; NA: Not Available.

**Table 3:** Examples of strengthening exercise [129].

**Quadriceps Strengthening Exercises**
1. Perform a supine straight leg lift, lifting the leg to 30° of hip flexion and applying resistance with ankle weights.
2. Execute a knee extension within a limited arc, utilizing a knee roll for stabilization and applying resistance using ankle weights.
3. Attain full knee extension in a seated position, starting from 90° of knee flexion, and introduce resistance through ankle weights.
4. Execute small arc squats on both legs, ranging from full extension to 30° of knee flexion, with a ball braced against a wall. Utilize two dumbbells, one in each hand.
5. Engage in small arc squats on both legs, ranging from 40° to 90° of knee flexion, with a ball against a wall. Apply resistance using two dumbbells, one in each hand.
6. Perform step-ups on a ‘stepper’ platform with a height of 30 cm.
**Hip Strengthening Exercises**
1. Side-lying Abduction: performing unilateral hip abduction while lying on one side, utilizing ankle cuff weights.
2. Standing Abduction: executing unilateral hip abduction in a standing position with the aid of a resistance band.
3. Isometric Hip Abduction against a Wall in Standing Position: conducted in a monopodial stance with the opposite limb at a 90° knee flexion.
4. Clam Exercise in Side-Lying Posture using an Elastic Band for Resistance.
5. Bilateral Bridging Exercise.
6. Unilateral Bridging with the opposite limb flexed at approximately 90° at the knee.

**Table 4:** Examples of neuromuscular exercise [127].

Neuromuscular Exercises
1. Standing on both feet on a cushioned surface.
2. Standing on both feet on a soft surface with closed eyes.
3. Two children standing and facing each other while throwing a ball, on a soft surface.
4. Stationary squat lunge on a cushioned surface.
5. Stationary squat lunge on a soft surface while tossing and catching a ball.
6. Balancing on one foot on a soft surface.
7. Balancing on one foot on a soft surface with closed eyes.

**Table 5:** Examples of flexibility exercise.

Flexibility Exercises
1. Trunk Flexion and Extension.
2. Hip Flexion and Extension.
3. Leg Flexion and Extension.

## Data Availability

Not applicable.
